# Open and Laparo-Endoscopic Repair of Incarcerated Abdominal Wall Hernias by the Use of Biological and Biosynthetic Meshes

**DOI:** 10.3389/fsurg.2016.00010

**Published:** 2016-02-25

**Authors:** René H. Fortelny, Anna Hofmann, Christopher May, Ferdinand Köckerling, BioMesh Study Group

**Affiliations:** ^1^Department of General, Visceral and Oncological Surgery, Wilhelminenspital, Vienna, Austria; ^2^Department of Surgery, Center for Minimally Invasive Surgery, Vivantes Hospital, Berlin, Germany

**Keywords:** incarceration, strangulation, groin hernia surgery, abdominal wall hernia, biological mesh, incisional hernia, ventral hernia, bio-resorbable mesh

## Abstract

**Introduction:**

Although recently published guidelines recommend against the use of synthetic non-absorbable materials in cases of potentially contaminated or contaminated surgical fields due to the increased risk of infection (1, 2), the use of bio-prosthetic meshes for abdominal wall or ventral hernia repair is still controversially discussed in such cases. Bio-prosthetic meshes have been recommended due to less susceptibility for infection and the decreased risk of subsequent mesh explantation. The purpose of this review is to elucidate if there are any indications for the use of biological and biosynthetic meshes in incarcerated abdominal wall hernias based on the recently published literature.

**Methods:**

A literature search of the Medline database using the PubMed search engine, using the keywords returned 486 articles up to June 2015. The full text of 486 articles was assessed and 13 relevant papers were identified including 5 retrospective case cohort studies, 2 case-controlled studies, and 6 case series.

**Results:**

The results of Franklin et al. (3–5) included the highest number of biological mesh repairs (Surgisis^®^) by laparoscopic IPOM in infected fields, which demonstrated a very low incidence of infection and recurrence (0.7 and 5.2%). Han et al. (6) reported in his retrospective study, the highest number of treated patients due to incarcerated hernias by open approach using acellular dermal matrix (ADM^®^) with very low rate of infection as well as recurrences (1.6 and 15.9%). Both studies achieved acceptable outcome in a follow-up of at least 3.5 years compared to the use of synthetic mesh in this high-risk population (7).

**Conclusion:**

Currently, there is a very limited evidence for the use of biological and biosynthetic meshes in strangulated hernias in either open or laparo-endoscopic repair. Finally, there is an urgent need to start with randomized controlled comparative trials as well as to support registries with data to achieve more knowledge for tailored indication for the use of biological meshes.

## Introduction

The BioMesh Study Group has set itself the task of identifying the best way to use biological meshes for various indications. The first step (toward achieving that goal) is to compile systematic reviews of different indications on the basis of the existing literature. The available literature sources will be evaluated in accordance with the Oxford Centre for Evidence-based Medicine-Levels of Evidence (March 2009). Next, based on the review findings, corresponding Statements and Recommendations are to be formulated in a Consensus Conference for the use of biological meshes regarding different indications. The findings of the Consensus Conference will then be summarized as a joint publication. This present publication is part of the project undertaken by the BioMesh Study Group.

Although recently published guidelines recommend against the use of synthetic non-absorbable materials in cases of potentially contaminated or contaminated surgical fields due to the increased risk of infection (1, 2), the use of bio-prosthetic meshes for abdominal wall or ventral hernia repair is still controversially discussed in such cases. Especially in these indications, bio-­prosthetic meshes have been recommended due to less susceptibility for infection and the decreased risk of subsequent mesh explantation. The greatest drawback of bio-prosthetics is still the high cost in comparison to synthetic non-absorbable meshes (2). Above all, there is a lack of evidence concerning the clinical efficacy of biologic over synthetic non-absorbable meshes (7). In the literature, wound infection rates after the use of biological meshes even in clean-contaminated fields are reported up to 40% (8, 9) and hernia recurrence rates up to 30%, respectively (10). On the other hand, the reports of Zafar et al. (11) regarding emergency surgery of incarcerated incisional hernia with associated bowel obstructions enrolling 60 patients by the use of permanent prosthetic meshes revealed an almost identically high percentage (31%) of wound complications in a retrospective study. The purpose of this review is to elucidate if there are any indications for the use of biological and biosynthetic meshes in incarcerated abdominal wall hernias based on the recently published literature.

## Methods

A literature search of the Medline database using the PubMed search engine, using the keywords (incarcerated hernia OR strangulated hernia OR inguinal hernia OR Groin hernia OR inguinal hernia OR ventral hernia OR incisional hernia AND biological mesh OR Biomesh OR Biological OR biosynthetic mesh AND open repair OR laparoscopic repair OR endoscopic repair) returned 486 articles up to June 2015. Titles and abstracts were searched for the use of biologic meshes in open and laparo-endoscopic repair of incarcerated/strangulated abdominal wall hernias. The full text of 486 articles was assessed, and 13 relevant papers were identified including 5 retrospective case cohort studies (9, 12–14), 2 case-controlled studies (5, 15) and 6 case reports (16–21). A summary of study demographics and characteristics is presented in Table 1 and the outcome data in Table 2. Qualitative assessment of all included studies was based on the Oxford Centre for Evidence-Based Medicine 2009 levels of evidence.

**Table 1:** **Summary of study demographics and characteristics**.

Reference	Study design	COI	Patient (*n*)	Mean age	Mean BMI	FU	LoE
Quartey et al. (16)	CR	NR	1	71	NR	5 months	4
Ueno et al. (15)	CCS	NR	20	60.1	NR	15.7 months	4
Xourafas et al. (12)	RCS	NR	51	59	29 > 30	22 months	3
Helton et al. (13)	RCS	NR	53	51	32	14 months	4
Giakoustidis et al. (17)	CR	No	1	53	NR	12 months	4
Shah et al. (9)	RCS	NR	58	57.2	33.8	12 months	4
Tsuda (22)	CR	No	1	33	38	6 weeks	4
Franklin et al. (5)	CCS	NR	116	58	NR	52 months	4
Han et al. (6)	RCS	No	63	57	29	43 months	4
Patton et al. (14)	RCS	NR	67	55	NR	10.6 months	4
Fallis et al. (18)	CR	NR	1	81	NR	6 months	4
Gooch et al. (19)	CR	No	1	38	NR	4 years	4
Pulido et al. (20)	CR	NR	1	70	NR	NR	4
Schiergens et al. (21)	CR	NR	1	32	NR	NR	4

*n, number; RCS, Retrospective Cohort Study; CCS, Case–Control Study; CR, case report; COI, conflict of interest; NR, no report; LoE, level of evidence (based on the Oxford Centre for Levels of Evidence 2009)*.

**Table 2:** **Outcome data**.

Reference	Mesh (*n*)	Meshtype	Meshposition	Fixation	Hernia type (*n*)	Incarceration/strangulation	Resection (bowel)	Recurrence (%)	Wound infection (%)
Quartey et al. (16)	1	Flex-HD^®^	Inguinal	NR	Inguinal	1	1	0	0
Ueno et al. (15)	20	Surgisis^®^	Underlay (17)	NR	Ventral (18)	3	NR	0	0
			Onlay (3)		Inguinal (2)				
Xourafas et al. (12)	51	AlloDerm^®^ (4)	Underlay	NR	Ventral	NR	51	22	22
		Surgisis^®^ (1)							
		Synthetic mesh (46)							
Helton et al. (13)	53	Surgisis^®^	IPOM (2)	Sutures	Ventral	NR	13	17	24
			Underlay (41)						
			Onlay (3)						
Giakoustidis et al. (17)	1	NR	NR	NR	Incisional	1	0	0	0
Shah et al. (9)	58	AlloDerm^®^ (29)	Onlay (10)	NR	Ventral	9	NR	27.9	19
		Permacol^®^ and CollaMend^®^ (5)	Underlay (21)						
		Surgisis^®^ and Strattice^®^ (24)	Bridging (27)						
Tsuda et al. (22)	1	Strattice^®^	IPOM	Sutures	Incisional	1	Omentum resection	0	0
				Titanium spiral tacks					
Franklin et al. (5)	133	Surgisis^®^	IPOM	Tacks	Inguinal (29)	32	17	5.2	0.7
					Incisional (57) umbilical (38)				
					Femoral (3) Parastomal (2) Spigelian (4)				
Han et al. (6)	63	AlloDerm^®^	IPOM	Sutures	Ventral (45)	63	33	15.9	1.5
					Incisional (18)				
Patton et al. (14)	67	AlloDerm^®^	Inlay (43)	Sutures	Ventral	10	NR	17.9	16
			Interlay (28)						
			Onlay (5)						
Fallis et al. (18)	1	Strattice^®^	Perineal bridge	Sutures	Perineal	1	1	0	NR
Gooch et al. (19)	1	Permacol^®^	Hiatal	Sutures	Hiatal	1	0	0	NR
Pulido et al. (20)	1	Flex HD^®^	Diaphragmatic	Sutures	Diaphragmatic	1	0	NR	NR
Schiergens et al. (21)	1	BioA^®^	Hiatal	NR	Diaphragmatic	1	0	0	0

*n, number; NR, no report*.

## Results

In the special case of an incarcerated recurrent Amyand’s hernia, the only paper concerning the use of a biological mesh was published by Quartey et al. (16). After appendectomy in an open approach, an acellular hydrated dermis (Flex HD^®^) was implanted with an uneventful postoperative follow-up to 5 months. In a review regarding Amyand’s hernia, Michalinos et al. (23) concluded that in case of proper treatment, including the use of meshes, the morbidity or mortality is not increased beyond that of a typical inguinal hernia repair. Similar conclusions can be found in the review of Köckerling et al. (24) with the statement: “The use of biological meshes in inguinal hernia repair especially in potentially contaminated fields is an alternative to the use of synthetic meshes with reasonable recurrence rates.”

In the case cohort study of Ueno et al. (15) including 2 inguinal and 18 ventral hernias – 3 with incarceration – patients were treated with Surgisis^®^ mesh implants. In a follow-up of 15.7 months, no infection or recurrence was detected.

The retrospective case–control study of Xourafas et al. (12) regarding the use of meshes in incarcerated ventral hernia repair with a simultaneous bowel resection included five patients (out of 51 in the mesh group) with the implantation in underlay technique using Alloderm^®^ in four cases and Surgisis^®^ in one case, respectively. The overall infection and recurrence rate (synthetic and biological meshes) was 22% in a follow-up of 22 months. The result of an univariate and a multivariate analysis detected a significant risk of increased postoperative infection in the mesh group, without separation regarding the type of mesh.

Helton et al. (13) reported in a retrospective case–control study of 13 patients treated with bowel resection due to incarceration or strangulation in ventral hernia by the use of Surgisis Gold^®^ in an open approach. The wound infection rate was 24% and the recurrence rate 17% in a follow-up of 14 months.

In a retrospective study of different bio-prosthetic materials in complex ventral hernia repair by Shah et al. (9) nine patients with incarceration (out of 58) were included. Different biological meshes were used (Alloderm^®^, CollaMend^®^, Permacol^®^, Surgisis^®^, and Strattice^®^). The overall recurrence rate was 27.9%, and surgical wound infections were detected in 19% in a follow-up of 1 year. The 17.2% of the meshes required explantation. Non-cross-linked porcine biologics were less likely to be explanted, but had higher recurrence rates compared to cross-linked porcine biologics and a higher infection rate compared to Alloderm^®^ (non-cross-linked human dermis).

Franklin et al. published a case–control study using porcine small intestinal submucosa mesh (Surgisis^®^) for laparoscopic IPOM repair of hernias in infected fields in the years 2002, 2004, and 2008 (3–5). In summary, 133 procedures were performed in 116 patients of which 17 (12.7%) required a bowel resection due to strangulated hernias with necrotic bowel. The overall recurrence rate was 5.2% and the infection rate 0.7% in a mean follow-up of 52 months.

Incarcerated abdominal wall hernias treated with the use of human dermal matrix (ADM^®^) in IPOM position by open approach in combination with vacuum wound drainage was reported by Han et al. (6) in a retrospective study. In 33 out of 63 incarcerated hernias, bowel resection was performed. In a follow-up of 43 months, 15.9% recurrences were detected and 1.6% suffered from a superficial wound infection. Multivariate analysis isolated BMI, defect size, and numbers of biological meshes used as risk factors to significantly affect recurrence rates.

Patton et al. (14) published a retrospective study of abdominal wall reconstructions with the use of acellular dermal matrix (ADM^®^) in complex and contaminated ventral hernias. In 51% of the repairs, the mesh was positioned as IPOM bridging with 3 cm overlap, 42% as an interlay, and 8% as an onlay. The 13 patients out of 89 were treated in case of incarcerated hernias. Overall, 16% developed wound infections, and in a follow-up of 10.6 months, 17.9% suffered from a recurrent hernia.

There are some single case reports like Giakoustidis et al. (17) reporting of a biological mesh used in an incarcerated recurrent incisional hernia as well as Tsuda (22) describing a laparoscopic repair of an incarcerated umbilical hernia using Strattice^®^ and Fallis et al. (18) publishing an open mesh repair of a strangulated perineal hernia after abdominoperineal resection. Another single case was reported by Gooch et al. (19) concerning a transthoracic repair of an incarcerated diaphragmatic hernia with a cross-linked porcine dermal collagen (Permacol^®^) and finally Pulido et al. (20) who described a laparoscopic repair in a case of chronic traumatic diaphragmatic hernia containing an obstructed small bowel and gallbladder also used Permacol^®^.

Schiergens et al. (21) reported of an emergent laparoscopic fundoplication of acute upside-down stomach with incarceration using biocompatible gradually absorbable synthetic polymers (BioA^®^) in a 32-year-old male patient. The follow-up was uneventful.

## Discussion/Summary

In summary, so far the data regarding the use of biological and biosynthetic meshes are very scarce and there is only one level 3 study published up to now. The results of this study of Xourafas et al. (12) comparing mesh versus mesh-free repair of ventral hernia with a simultaneous bowel resection obtained a significant risk factor for the mesh group concerning the development of an infection. On multivariate regression analysis, the risk was present irrespective of drain use, defect size, and type of bowel resection. However, the analysis of a subgroup of 10 patients treated with the use of biological meshes out of a total of 100, which underwent mesh repair, did not reveal a single infection, whereas the group of polypropylene meshes showed a 24% infection rate. There was no reported significant difference in the incidence of recurrences between the mesh- and the mesh-free group (22 versus 24%), but unfortunately no comparative analysis between synthetic and biological meshes was published.

The results of Franklin et al. (3–5) include the highest number of biological mesh repairs (Surgisis^®^) in infected fields by laparoscopic approach, which demonstrated a low ratio of required bowel resection (12.7%), furthermore the overall incidence of recurrence and infection was very low (5.2 and 0.7%) in a mean follow-up of 52 months. Han et al. (6) reported, in his retrospective study, the highest number of treated patients due to incarcerated hernias with bowel resection by open approach using acellular dermal matrix (ADM^®^) with very low rate of infection (1.6%) as well as recurrences (15.9%) in a follow-up of 43 months. Both studies achieved acceptable outcome in a follow-up of at least 3.5 years compared to the use of synthetic mesh in this high-risk population (7).

In the conclusion of a critical review of biologic mesh use in ventral hernia repairs under contaminated conditions by Primus and Harris (7) as well as in the systematic review by Lee et al. (25), a similar statement can be found: “The available evidence is limited, but does not support the superiority of biologic over synthetic non-absorbable prosthetics in contaminated fields. Due to a lack of scientific evidence concerning the use of biologic mesh in case of laparoscopic treatment in incarcerated/strangulated ventral hernias (in potentially contaminated field) no recommendation or suggestion can be stated.”

Taking into account that there is a significantly increasing rate of emergent incisional hernia repair in the group of older men (>65 years) when analyzing the years 2001 to 2010 in the United States (26), the importance to treat this growing population with an appropriate method including the selection of mesh type and material should be addressed in further studies and registries. The results of a survey of practicing surgeons (members of American College of Surgeons) concerning the use of biological meshes in abdominal reconstructions (27) revealed a lack of consensus in terms of indication, surgical techniques, as well as type of biological mesh.

Looking to the different Guidelines based on consensus conferences of the European Association for Endoscopic Surgery (EAES), International Endo Hernia Society (IEHS), European Hernia Society (EHS), and the World Society of Emergency Surgery (WSES) (28–33), we can only find a recommendation of the WSES in terms of the question which kind of mesh should be used in incarcerated/strangulated hernia. The WSES guideline based on a Consensus Meeting in 2013 (29) recommends the use of biological meshes as a valid option in case of emergency hernia repair in potentially contaminated surgical field for patients with intestinal strangulation and/or concurrent bowel resection [grade 2C recommendation GRADE (34)]. In case of stable patients with strangulated obstruction and peritonitis by bowel perforation (contaminated-dirty surgical field), direct tissue suture is recommended when the hernia defect is small; in the events that direct tissue suture is not possible, biological mesh repair may be suggested (grade 2C recommendation). The choice between cross-linked and non-cross-linked biological mesh should be evaluated depending on the defect size and degree of contamination (grade 2C recommendation).

Without any doubt, currently there is a very limited evidence for the use of biological and biosynthetic meshes in strangulated hernias in open as well as in laparo-endoscopic repair. Finally, there is an urgent need to start with randomized controlled comparative trials as well as to support registries with data to achieve more knowledge for tailored indication for the use of biological meshes.

## Author Contributions

RF main authorship, corresponding author. AH support in selection of papers of the review, composing tables of the manuscript. CM support in composing of the manuscript, proof reading. FK final proof.

## BioMesh Study Group

Ferdinand Köckerling (Chairman), Stavros Antoniou, René H Fortelny, Frank A. Granderath, Markus Heiss, Franz Mayer, Marc Miserez, Agneta Montgomery, Salvador Morales-Conde, Filip Muysoms, Alexander Petter-Puchner, Rudolph Pointner, Neil Smart, Marciej Smietanski, Bernd Stechemesser.

## Conflict of Interest Statement

The authors declare that the research was conducted in the absence of any commercial or financial relationships that could be construed as a potential conflict of interest.
